# 45 Endothelial Damage Occurs Early After Inhalation Injury as Measured by Increased syndecan-1 Levels

**DOI:** 10.1093/jbcr/irac012.048

**Published:** 2022-03-23

**Authors:** Edward J Kelly, John W Keyloun, Bonnie C Carney, Lauren T Moffatt, Jeffrey W Shupp

**Affiliations:** Medstar Washington Hospital Center, Alexandria, Virginia; Burn Center at MedStar Washington Hospital Center, Washington DC, District of Columbia; Medstar Health Research Institute, Washington DC, District of Columbia; Burn Center at MedStar Washington Hospital Center, Washington DC, District of Columbia; MedStar Washington Hospital Center, Washington DC, District of Columbia

## Abstract

**Introduction:**

Inhalation injury is a significant cause of morbidity and mortality in the burn patient population. However, the pathogenesis of inhalation injury and its potential involvement in burn shock is not well understood. Pre-clinical studies have shown endothelial injury, as measured by syndecan-1 levels, to be involved in the increased vascular permeability seen in shock states. Furthermore, the lung has been identified as a site of significant syndecan-1 shedding. Here we aim to characterize the contribution of endotheliopathy caused by inhalation alone in a swine model.

**Methods:**

Eight female Yorkshire pigs were used in this experiment. A custom-made smoke box was employed to deliver smoke via endotracheal tube directly into the swine lungs. Carboxyhemoglobin levels were then titrated to a level of 50-75%. Blood was collected at induction of anesthesia, pre-injury, 30 minutes, and at hours 1, 2, 4, 6, and 12 post-injury and was stored in EDTA tubes from which plasma was separated and stored for future analysis. Pigs were necropsied immediately after completion of the experiment and lung samples were placed in all-protect and flash frozen. Histology was performed on lung sections and a validated, published scoring system composed of 5 parameters (neutrophils in the alveolar space, neutrophils in the interstitial space, hyaline membrane formation, protein detritus in the alveolar space and septum thickening) was used to assess lung injury severity (between 0 and 1).Plasma Syndecan-1 (SDC-1) was quantified by ELISA. All data was compared to Syndecan-1 levels measured at induction. Conditions were analyzed with one-way ANOVA with multiple comparisons and Dunnett’s correction for multiple comparisons.

**Results:**

Syndecan-1 levels at induction were 13.74 ± 2.03 ng/ml. Pre-injury and 30 minutes post-injury levels remained similar. Syndecan-1 levels at hour 2 post-injury increased 37% from induction (18.36 ± 1.28 ng/ml, p=0.0057). This trend continued with a 47% percent increase from induction at hour 4 post-injury (19.62 ± 2.15 ng/ml, p=0.0033) and a 49% increase from induction at hour 6 post-injury (20.42 ± 2.43 ng/ml, p=0.0011). Histological sections showed higher lung injury severity compared to control pigs (0.1-0.3 vs. 0.5-0.74, p< .05).

**Conclusions:**

Significant increases in syndecan-1 levels in this animal model provide evidence for a connection between smoke inhalation injury and endothelial injury. Furthermore, the endotheliopathy that leads to burn shock could be exacerbated by inhalation injury, leading to the poor clinical outcomes that are often seen in patients with combined burn and inhalation injuries. Future research should focus on the mechanisms underlying inhalation injury and its contribution to shock physiology.

